# Facility based cross-sectional study of self stigma among people with mental illness: towards patient empowerment approach

**DOI:** 10.1186/1752-4458-7-21

**Published:** 2013-09-03

**Authors:** Eshetu Girma, Markos Tesfaye, Guenter Froeschl, Anne Maria Möller-Leimkühler, Sandra Dehning, Norbert Müller

**Affiliations:** 1 Department of Health Education and Behavioral Sciences, Jimma University, Jimma, Ethiopia; 2 CIHLMU Center for International Health, Ludwig-Maximilians-Universität, Munich, Germany; 3 Department of Psychiatry, Jimma University, Jimma, Ethiopia; 4 Department of Infectious Diseases and Tropical Medicine, Ludwig-Maximilians-Universität, Munich, Germany; 5 Department of Psychiatry and Psychotherapy, Ludwig-Maximilians-Universität, Munich, Germany

**Keywords:** Self stigma, Internalized stigma, Stigma, Mental illness, People with mental illness

## Abstract

**Background:**

Self stigma among people with mental illness results from multiple cognitive and environmental factors and processes. It can negatively affect adherence to psychiatric services, self esteem, hope, social integration and quality of life of people with mental illness. The purpose of this study was to measure the level of self stigma and its correlates among people with mental illness at Jimma University Specialized Hospital, Psychiatry clinic in southwest Ethiopia.

**Methods:**

Facility based cross-sectional study was conducted on 422 consecutive samples of people with mental illness using interviewer administered and pretested internalized stigma of mental illness (ISMI) scale. Data was entered using EPI-DATA and analysis was done using STATA software. Bivariate and multivariate linear regressions were done to identify correlates of self stigma.

**Results:**

On a scale ranging from 1 to 4, the mean self stigma score was 2.32 (SD = 0.30). Females had higher self stigma (std. β = 0.11, P < 0.05) than males. Patients with a history of traditional treatment had higher self stigma (std. β = 0.11, P < 0.05). There was an inverse relationship between level of education and self-stigma (std. β = −0.17, P < 0.01). Perceived signs (std. β = 0.13, P < 0.05) and supernatural causes of mental illness (std. β = 0.16, P < 0.01) were positively correlated with self stigma. Higher number of drug side effects were positively correlated (std. β = 0.15, P < 0.05) while higher self esteem was negatively correlated (std. β = −0.14, P < 0.01) with self stigma.

**Conclusions:**

High feeling of inferiority (alienation) but less agreement with common stereotypes (stereotype endorsement) was found. Female showed higher self stigma than male. History of traditional treatment and higher perceived supernatural explanation of mental illness were associated with higher self stigma. Drug side effects and perceived signs of mental illness were correlated with increased self stigma while education and self esteem decreased self stigma among people with mental illness. Patient empowerment psychosocial interventions and strategies to reduce drug side effects can be helpful in reducing self stigma among people with mental illnesses.

## Background

Stigma against people with mental illness is a complex public health problem which exists in different forms and many actors like the public, family members, media, patients themselves and even sometimes the health providers are involved [1-3]. Studies indicated that public stigma against people with mental illness is highly associated with self stigma among the patients [4,5]. Since self stigma can also exist without actual stigma from the public, more hidden and inside, it seems to be the worst form of stigma against people with mental illness and can directly affect the patients over all well being [6]. For example, researchers have shown that self stigma among people with mental illness affects adherence to psychiatric services, self esteem, hope and quality of life negatively [7-10]. Moreover, it is also a great barrier for social integration [6]. On the other hand, social integration is usually reported to be one of the most effective strategies for reducing both self and public stigma against people with mental illness [11]. Generally, it is a result of multiple cognitive and environmental processes and factors.

When people with mental illness bear high level of self stigma, they may have less resistance capability to public stigma, and thus submissive to discriminatory behaviors so that it negatively affects the rehabilitation and treatment processes of the patients. For instance, more than two thirds of people with mental illness in England reported that they have stopped doing things they wanted to do because of self stigma. Two thirds of people with mental health problems live alone about four times more than the general population [12].

Systematic reviews of stigma identified that combating wrongly held beliefs about mental illness, improving self-esteem, empowerment (education), help seeking behavior, protesting stigma and advocacy for mental health as the most important self stigma reduction strategies concerning the patients. In these reviews, targeting high risk groups was suggested to combat self stigma among people with mental illness [13,14]. High delay in treatment seeking for mental illness was reported among Jimma University specialized hospital (where the current study was conducted) mental illness attendants [15] which might be attributed to self stigma.

Studies conducted using the internalized stigma of mental illness (ISMI) scale in Europe and Iran reported high prevalence of self stigma among people with schizophrenia [16,17]. A study in the capital city of Ethiopia on outpatients with schizophrenia using the same scale also reported high prevalence of self stigma [18]. In the above study, patients who were living in rural areas were more likely to exhibit higher self stigma than urban residents. Being single as marital status also predicted higher self stigma. Patients with psychotic symptoms scored significantly higher self stigma [18].

Those patients who receive modern psychiatric treatment are expected to have lower self stigma if they pass through a systematic psychosocial approach beside the biomedical treatment model process. But the level and correlates of self stigma among new and follow-up psychiatric patients in southwest Ethiopia particularly in Jimma University Specialized Hospital (JUSH) Psychiatry clinic attendants has not been investigated. The main purpose of this study was hence, to measure the level of self stigma and its correlates among JUSH, Psychiatry clinic attendants of people with mental illness in southwest Ethiopia.

## Methods

### Study design and setting

Hospital based cross-sectional study was conducted from June to August 2012 in Jimma University specialized hospital (JUSH) among psychiatric service attendants. JUSH is a teaching and referral hospital located in Jimma city 352 km southwest of Addis Ababa, Ethiopia. Each year, the hospital serves for approximately 9, 000 inpatients and 80,000 outpatients with a catchment population of about 15 million [19]. Psychiatry is among the 15 clinical services in the hospital serving psychiatric patients coming from Jimma area as well as patients referred from other health institutions in the southwestern region of the country. Over one thousand outpatients receive psychiatric care monthly. It also provides inpatient and outreach services [20].

### Sampling procedure

Representative sample of 422 consecutive new and follow-up psychiatric services attendants were included in this study. The sample size was determined using single population proportion formula by assuming 50% level of self stigma to get the maximum sample size, at 95% confidence level and considering and a 5% margin of error and non-response contingency. Respondents were screened using the Clinical Global Impression (CGI) scale to assess their eligibility to participate in the interview for the study [21]. The scale assesses the degree of the severity of the patients’ mental illness, improvement of their illness and efficacy index of therapeutic and drug side effects. New patients were screened only for the severity of their illness. Using this scale and their clinical experience, the psychiatric nurses identified the eligible respondents. Patients who were severely psychotic, incoherent and too disorganized to engage in the interviews of the study were excluded. Therefore, patients included in the study were only those who were above 18 years old and rated with at least a less severe state of mental illness, on improvement and good efficacy index by the psychiatry nurses.

### Data collection procedure

Data was collected by trained psychiatric nurses at JUSH, Psychiatry clinic through interviewer-administered questionnaires and a patient chart review to identify their diagnosis and other medical information. The data collection was supervised by specialist mental health workers. Data collectors and the supervisors were trained on the contents and procedures of the data collection.

### Measurement

To measure self stigma among the patients, the Internalized Stigma of Mental Illness (ISMI) Scale [22] was used. The scale has been used in several studies [16-18,22] . The ISMI scale have a total of 29 items on a 4-point Likert (1 = strongly agree to 4 = strongly disagree) measure containing five subscales; Alienation (6 items), Stereotype Endorsement (7 items), Discrimination Experience (5 items), Social Withdrawal (6 items), and Stigma Resistance (5 items). **Alienation** is “the subjective experience of being less than a full member of society*”*. The **Stereotype Endorsement** is “the degree to which patients agreed with common stereotypes about people with a mental illness”. The **Discrimination Experience** measures “respondents’ perceptions of the way they tend to be treated by others”. The **Social Withdrawal** measures the self exclusion from social events/situation due to mental illness”. The **Stigma Resistance** subscale is “a person’s ability to resist stigma” [17]. Unlike the above four subscales, higher score in this subscale indicated lower stigma resistance. Overall **self stigma** score was obtained by summing the scores of the five subscales. Higher score showed higher self stigma.

A study in Iran showed that the ISMI subscales had reliability values (Cronbach’s alpha) of (alienation = 0.84, stereotype endorsement = 0.71, discrimination experience = 0.87, social withdrawal = 0.85 and stigma resistance = 0.63). In the current study, the following reliability values (Cronbach’s alpha) were found: alienation = 0.84, stereotype endorsement = 0.73, discrimination experience = 0.79, social withdrawal = 0.77, stigma resistance = 0.65, over all self stigma = 0.89.

In addition to the ISMI scale, **self esteem** was measured using the Rosenberg self-esteem scale [23]. The scale has 10 Likert scale items with possible scores of 1 = strongly agree to 4 = strongly disagree. Higher score indicated higher self esteem. In addition, checklist was used to extract relevant data on the diagnosis and other medical information or data (example: co-morbidity and drug side effects) from the patients’ charts in the clinic. The questionnaire also included socio-demographic and psychographic characteristics related to mental illness (example: perceived causes and signs of mental illness and exposure to mental illness information).

The whole questionnaire was translated and administered in local languages (Affan Oromo and Amharic) and it was back translated to English to ensure semantic equivalence. The questionnaire was also pre-tested in the psychiatric clinic before the main study. Based on the pre-test, some items were modified and more clarifications were given to the data collectors on items which were not understood well.

### Statistical analysis

After checking for the completeness of each questionnaire, data entered was done using EPI-DATA version 3.1 and then exported to STATA version 10.0 for analysis. A frequency table was computed for socio-demographic and other variables. Stigma scores were checked for normal distribution. Tests of significant mean differences (*t* test and ANOVA) of stigma scores and other variables were done for each of the five subscales of ISMI separately and for the overall self stigma scores. Six separate multivariate linear regression models were developed using variables which had significant statistical associations with the respective subscales and the overall self stigma scores during bivariate analysis. Unadjusted and adjusted standardized regression coefficients were presented for each variable in each model. A P-value <0.05 was used to declare significant statistical association. Multicollinearity between variables was checked using tolerance analysis (variance inflation factor).

### Ethical approval

Ethical approval was secured from Jimma University Research Ethics Review Board. Written permission was obtained from JUSH clinical director and the Psychiatry clinic. Written informed consent was also obtained from each study participant.

## Results

### Background characteristics

Of the total 422 respondents, 227 (53.79%) were urban residents. Two hundred and ninety six (70.14%) of the respondents were male. The mean age was 33.11 (SD = 11.37) years. Two hundred and nine (49.53%) of them were single in marital status. Majority were Muslim religion followers (59.24%) and Oromo ethnic groups (60.43%). One hundred and eighty six (44.08%) of them were in secondary educational status and majority were farmers (28.44%) and private enterprise workers (25.12%). Average family size was 5.37 (SD = 2.72). The average family monthly income was about 74.70 (SD = 120.15) USD (Table 1).

**Table 1:** Background characteristics of people with mental illness in Jimma University specialized hospital, Southwest Ethiopia, 2012

**Characteristics**	**Frequency**	**Percent**
**Sex**		
Male	296	70.14
Female	126	29.86
**Marital status**		
Single	209	49.53
Married	183	43.36
Divorced and widowed	30	7.11
**Religion**		
Muslim	250	59.24
Orthodox	116	27.49
Others (Protestant, Catholic, Waqefeta)	56	13.27
**Ethnicity**		
Oromo	255	60.43
Amhara	64	15.17
Others (Keffa, Dawro, Gurage)	103	24.41
**Educational status**		
Could not read and write	45	10.66
Read and write only	37	8.77
Elementary	83	19.67
Secondary	186	44.08
Higher education	71	16.82
**Occupation**		
Farmer	120	28.44
Private enterprise	106	25.12
Government employee	80	18.96
Student	57	13.51
Others (housewife and unemployed)	59	13.98
**Setting**		
Rural	195	46.21
Urban	227	53.79

### Diagnosis, perception and experiences

The majority of the patients were diagnosed with mood (49.05%) and psychotic (36.02%) disorders. The remaining 9.00% and 5.92% were diagnosed with anxiety and other disorders (substance related and personality disorders) respectively. Beside their psychiatric diagnosis, 19 (4.50%) had co-morbidities and 194 (45.97%) reported some kind of side effects of their medication. In addition, the health providers identified a mean of 2.53 (SD = 0.97) number of side effects attributed to the patients’ medications. The mean time since the onset of the patients mental illness was 5.87 (SD = 4.80) years while the mean time since the start of medical follow up was 4.55 (4.25) years. Mean number of visits to the psychiatric hospital was 21.51 (SD = 22.56). Two hundred and sixteen (51.18%) ever had experience of traditional treatment before seeking help at the psychiatric clinic.

**Figure 1:**
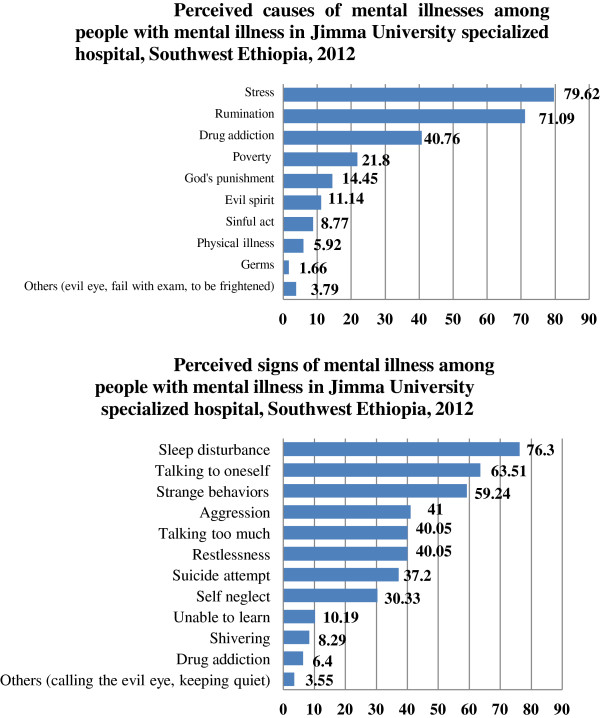
Percentage distribution of perceived causes and perceived signs of mental illnesses among people with mental illness in Jimma University specialized hospital, Southwest Ethiopia, 2012.

Seventy six (18.01%) had family/relative with a history of mental illness episodes. Regardless of the contents of the messages, 16.59%, 15.17% and 3.08% watched and heard about mental illness on television, radio and in religious places respectively in the period of one year before the time of data collection. Stress, rumination and drug addiction were the leading perceived causes of mental illness and sleep disturbance, talking to oneself and showing strange behaviours were the top three perceived signs of mental illnesses (Figure 1). Majority of the respondents, 407 (96.45%) believed that mental illness can be cured. The mean self esteem score was 2.68 (SD = 0.27).

### Stigma scores and their correlates

For each of the five subscales of ISMI and the overall self stigma scores a separate linear regression multivariate models were developed by entering variables which had significant statistical associations with the respective subscales and the overall self stigma scores during bivariate analysis.

### Alienation

Out of a four point scale, the mean alienation (feeling of being inferior) score was 2.46(SD = 0.50). Females had significantly higher alienation (std. β = 0.11, P < 0.05) than males. Those patients who ever had traditional treatment had also higher alienation (std. β = 0.15, P < 0.01). Higher education level was significantly correlated with lower alienation (std. β = −0.18, P < 0.001) while higher scores of perceived supernatural causes of mental illness was significantly correlated with increased alienation (std. β = 0.11, P < 0.05). As the duration treatment increased, alienation score decreased significantly (std. β = −0.12, P < 0.05). This model explained 15% of the variance of alienation.

### Stereotype endorsement

The mean score for agreeing on the common stereotypes about people with a mental illness was 2.20 (SD = 0.34). Compared with farmers, private enterprise workers (std. β = −0.23, P < 0.01), government employees (std. β = −0.14, P < 0.05) and students (std. β = −0.18, P < 0.01) had significantly lower stereotype endorsement scores. Patients with higher education (std. β = −0.16, P < 0.01) and higher self esteem (std. β = −0.11, P < 0.05) had lower stereotype endorsement. Higher perceivement of supernatural causes of mental illness was correlated with higher stereotype endorsement (std. β = 0.16, P < 0.01). The model explained 13% of the variance in stereotype endorsement.

### Discrimination experience

Mean perceived discrimination score was 2.28 (SD = 0.42). As education level increases, discrimination experience score decreases significantly (std. β = −0.13, P < 0.05). Respondents with higher score in perceived supernatural causes (std. β = 0.13, P < 0.01) and higher number of drug side effects (std. β = 0.16, P < 0.01) had higher discrimination experience scores. The explained variance of this model was 10%.

### Social withdrawal

Mean score for self exclusion from social events was 2.27 (SD = 0.38). Experiences with traditional treatment were significantly associated with an increase in social withdrawal score (std. β = 0.14, P < 0.01). Significant decrease in social withdrawal was observed as the educational status of individuals increased (std. β = −0.11, P < 0.05) while there was a significant increase in social withdrawal when the score in perceived supernatural causes of mental illness increased (std. β = 0.13, P < 0.01). The model explained only 7% of the variance in social withdrawal.

### Stigma resistance

The mean score for stigma resistance subscale was 2.41 (SD = 0.40). Patients with substance related disorders and personality disorders (std. β = −0.13, P < 0.01) had significantly better stigma resistance than patients with diagnosis of mood disorder. Patients with higher education (std. β = −0.10, P < 0.05) and higher self esteem (std. β = −0.40, P < 0.001) had better stigma resistance compared with their counterparts. This model explained 20% of the variance in stigma resistance (Table 2).

**Table 2:** Determinants of self stigma subscales among people with mental illness in Jimma University Specialized hospital, Southwest Ethiopia, 2012

**Subscale**	**Unadjusted β (standardized)**	**Adjusted β (standardized)**
**Alienation**		
Female	0.15^**^	0.11^*^
Ever had traditional treatment	0.20^***^	0.15^**^
Any drug side effect	0.20^***^	0.03
Education	−0.16^**^	−0.18^***^
Perceived signs of mental illness	0.21^***^	0.14^*^
Perceived supernatural causes	0.16^**^	0.10^*^
Perceived psychosocial and biological causes	0.14^**^	0.04
Duration of start of treatment	−0.11^*^	−0.12^*^
Number of drug side effects	0.24^***^	0.11
**Stereotype endorsement**		
Private enterprise *(reference = farmers)*	−0.01	−0.23^**^
Government employee *(reference = farmers)*	−0.11	−0.14^*^
Student *(reference = farmers)*	−0.32^***^	−0.18^**^
Others *(reference = farmers)*	−0.28^***^	−0.11
Urban	−0.15^**^	0.08
Education	−0.26^***^	−0.16^**^
Perceived supernatural causes	0.19^***^	0.16^**^
Exposure to mental illness information	−0.14^**^	−0.07
Duration of start of treatment	−0.11^*^	−0.06
Self esteem	−0.15^**^	−0.11^*^
**Discrimination experience**		
Any side effect	0.20^***^	0.07
Education	−0.10^*^	−0.13^*^
Perceived signs	0.21^***^	0.13^*^
Perceived supernatural causes	0.18^***^	0.13^**^
Perceived psychosocial and biological causes	0.12^*^	−0.01
Number of drug side effects	0.24^***^	0.16^**^
**Social withdrawal**		
Ever had traditional treatment	0.18^***^	0.14^**^
Any side effects	0.14^**^	0.07
Education	−0.12^*^	−0.11^*^
Perceived supernatural causes	0.16^**^	0.13^**^
Number of drug side effects	0.14^**^	0.06
**Stigma resistance**		
Anxiety disorders *(reference = Mood disorders)*	−0.04	−0.07
Psychotic disorders *(reference = Mood disorders)*	−0.06	−0.04
Others (substance use and personality disorders) (*reference = mood disorders)*	−0.14^**^	−0.13^**^
Education	−0.13^**^	−0.10^*^
Perceived supernatural causes	0.15^**^	0.12^**^
Self esteem	−0.42^***^	−0.40^***^

^*^P < 0.05, ^**^P < 0.01, ^***^P < 0.001.

### Overall self stigma

The overall self stigma mean score was 2.32 (SD = 0.30). Among the total respondents, 25.12% of them showed 2.5 and above self stigma score. Compared with males, females had higher self stigma (std. β = 0.11, P < 0.05). Private enterprise workers had significantly lower self stigma (std. β = −0.15, P < 0.05) than farmers. Patients who ever had traditional treatment had higher self stigma (std. β = 0.11, P < 0.05) than patients without a history of traditional treatment. Higher education was significantly correlated with lower self stigma (std. β = −0.17, P < 0.01). Increase in perceived signs (std. β = 0.13, P < 0.05) and perceived supernatural causes of mental illness (std. β = 0.16, P < 0.01) was significantly correlated with an increase in self stigma among patients with mental illness. Higher number of drug side effects positively correlated (std. β = 0.15, P < 0.05) while higher self esteem negatively correlated (std. β = −0.14, P < 0.01) with self stigma. The multivariate model explained 18% of the variance in self stigma among people with mental illness (Table 3).

**Table 3:** Determinants of self stigma among people with mental illness in Jimma University Specialized hospital, Southwest Ethiopia, 2012

**Variable**	**Unadjusted β (standardized)**	**Adjusted β (standardized)**
Female	0.10^*^	0.11^*^
Private enterprise *(reference = farmers)*	−0.19^**^	−0.15^*^
Government employee *(reference = farmers)*	−0.12^*^	−0.05
Student *(reference = farmers)*	−0.11^*^	−0.08
Others *(reference = farmers)*	−0.08	−0.10
Ever had traditional treatment	0.17^**^	0.11^*^
Any side effects	0.16^**^	0.02
Education	−0.21^***^	−0.17^**^
Perceived signs	0.18^***^	0.13^*^
Perceived supernatural causes	0.23^***^	0.16^**^
Perceived psychosocial and biological causes	0.10^*^	−0.01
Duration of start of treatment	−0.11^*^	−0.08
Number of drug side effects	0.22^***^	0.15^*^
Self esteem	−0.15^**^	−0.14^**^

^*^P < 0.05, ^**^P < 0.01, ^***^P < 0.001.

## Discussion

Compared with other studies using ISMI scale in Iran, Europe, USA and Ethiopia [16-18,24], a lower score of self stigma was found in this study. This could be attributed to the difference in the severity of mental illness since all the above mentioned studies were conducted only among patients with schizophrenia while the current study was conducted among patients from mild to severe mental health problems. In addition, based on the CGI screening test, patients with more severe state of illness and not able to take part in the interviews as a result, were excluded from the study which might have resulted in an obvious selection bias to the study. The fact that self stigma did not significantly differ among patients with different diagnosis in the current study might be also due to the selection bias.

Similar to a study in Europe [16], the present results indicated high feelings of inferiority (alienation) but less agreement with common stereotypes (stereotype endorsement) about people with mental illness scores. Especially, females, those who ever used traditional treatment and had higher perceived supernatural causes scored significantly higher on feelings of inferiority (alienation). This could be caused by the fact that anti-stigma interventions might be targeted at only tackling the common stereotypes from the community without much emphasis on positive self feelings and image development or empowerment processes. Furthermore, these groups might have been exposed to more blaming explanation of mental illness and social disadvantages. To this point, for example, there was no statistically significant difference in self stigma with regard to frequency of hospital visit as well as duration of treatment in the hospital. These segments of the participants had not only scored higher in alienation subscale but also they have shown significantly higher results in the overall self stigma score. A possible explanation might be that less stereotype endorsement could be due to less awareness of people with mental illness about the common stereotypes held within their community [25].

No statistical difference was observed with regard to religion, ethnicity, setting (urban/rural), marital status, age and income status. These factors were usually identified as important predictors of stigma in other studies [17,18,24,26]. One possible explanation for why such cultural and social domains did not explain self stigma may be that most respondents were more educated and had psychosocial explanation of mental illness. Similar to a study conducted in 13 European countries [27], data of the present study indicate that a higher educational level of the patients is significantly associated with lower scores in overall self stigma as well as in all five subscales of the ISMI. Education turned out to be the most powerful predictor of self stigma.

In contrast to the educational status of the patients, those individuals with higher perceived supernatural explanation of mental illness had significantly higher overall self stigma and higher scores in all the five subscales. Such association could have existed since patients with high perceived supernatural causes of mental illness may have had more self blaming explanation or that such patients possibly attended to western treatment in the hospital after trials and exhaustion of unsuccessful traditional and religious healings. Similarly, a higher score of perceived sign of mental illness were associated with higher alienation and discrimination experience subscales and overall self stigma scores. In addition, as the number of drug side effects increased, there was a significant increase in discrimination experience subscale and overall self stigma. These positive associations of higher perceived signs and number of drug side effects with self stigma can be related to the visible nature of the perceived signs and drug side effects (such as, weight gain, shaky hands, etc.) to other people.

The inverse relationship between self esteem and self stigma was reported in previous studies [7-9,28] and when we talk of self stigma, it is more or less directly or indirectly related with self esteem. In line with the above mentioned literature, a significant inverse relationship was found between self esteem on the one hand, and stereotype endorsement, stigma resistance and the overall self stigma scores on the other hand. Generally, compared with a study in a community hospital in Chicago, USA [25], the self esteem score obtained in this study was lower. As discussed above, this could be related to the general approach of fighting stigma by focusing on challenging the common public misconceptions and biomedical treatment without much emphasis on patient empowerment psychosocial approaches. Previous intervention suggested that patient empowerment approach is effective in reducing self stigma on Schizophrenia patients [29]. Because our study was conducted in a psychiatric facility and the data collectors were psychiatric nurses, there may be social desirability bias in the response of the patients. The patients who presented to the psychiatric facility might be those with lower self stigma and higher treatment seeking behavior, a fact representing a potential selection bias and limiting the potential to extrapolate this finding to patients who remained in the community.

## Conclusions

High feeling of inferiority (alienation) but less agreement with common stereotypes (stereotype endorsement) about people with mental illness was found. Females showed higher self stigma than males. History of traditional treatment and higher perceived supernatural explanation of mental illness were associated with higher self stigma. An increased educational status was one of the important factors which was inversely related to self stigma among people with mental illnesses. Higher number of drug side effects and perceived signs of mental illness were significant predictors of higher self stigma while high self esteem was correlated with lower self stigma. Psychosocial patient empowerment interventions with stronger emphasis on females, who ever had traditional treatment and who keep supernatural explanations of mental illness and who have les education, is recommended. Strategies which can reduce drug side effects can be helpful in reducing self stigma among people with mental illnesses. Further studies needs to be done whether self stigma is attached to gender roles.

## Competing interests

The authors declare that they have no competing interests.

## Authors’ contributions

EG, MT and SD designed the study, involved in the data collection, analysis and drafting of the manuscript. GF, AML, NM were involved in the design of the study, analysis of the data and critically reviewed the manuscript. All authors read and approved the final manuscript.
